# REGENERATION OF AGING LUNG TISSUE: A NOVEL WAY OF REOPENING THE PLASTICITY WINDOW OF POSTNATAL ALVEOLAR TYPE II CELLS

**DOI:** 10.1093/geroni/igac059.2673

**Published:** 2022-12-20

**Authors:** Hannah Hausman, Hannah Golding, Amitoj Sawhney, Douglas Brownfield

**Affiliations:** St. Catherine University, St. Paul, Minnesota, United States; University of Colorado Boulder, Boulder, Colorado, United States; Mayo Clinic, Rochester, Minnesota, United States; Mayo Clinic, Rochester, Minnesota, United States

## Abstract

Prevalence of chronic lung diseases, such as pulmonary fibrosis and chronic obstructive pulmonary disease, have been found to increase with age. Pulmonary disease can occur from failure to maintain alveolar type (AT) 1 and alveolar type 2 cells located in the epithelium of the lung. It is currently known that AT2 cells are the facultative stem cells of the lung, and can transform into AT1 cells which make up the structure of the alveoli. This process happens throughout embryonic development, but AT2 cells can also replenish AT1 cell populations post lung injury. This research investigates whether this process that normally occurs during development can also occur in mature AT2 cells, and studies this question using genetically engineered mouse models at a postnatal stage. Using a double inhibition mechanism, we attempted to increase cellular proliferation by administering an agonist and conducted cellular lineage tracing with an SpcCreER lox system via tamoxifen-induced GFP expression. Through lineage labeling, we saw AT1 cells arising from AT2 cells that became plastic, mirroring the plasticity observed in development. Further research is needed to determine if this agonist can be used to epigenetically unlock mature AT2 cells. This result would provide a mechanism to temporarily induce plasticity in a patient’s own AT2 cells in an attempt to regenerate their lungs in disease.

